# Digital Health Interventions for Quality Improvements in Chronic Kidney Disease Primary Care: A Systematic Review and Meta-Analysis of Randomized Controlled Trials

**DOI:** 10.3390/jcm13020364

**Published:** 2024-01-09

**Authors:** Miao Hui, Duoduo Zhang, Lili Ye, Jicheng Lv, Li Yang

**Affiliations:** 1 Renal Division, Department of Medicine, Peking University First Hospital, Beijing 100034, Chinajichenglv@bjmu.edu.cn (J.L.); liyang@bjmu.edu.cn (L.Y.); 2 Department of Cell Biology and Stem Cell Research Center, School of Basic Medical Sciences, Peking University Health Science Center, Beijing 100191, China; 3 Key Laboratory of Renal Disease, National Health Commission of China, Beijing 100191, China; 4 Key Laboratory of Chronic Kidney Disease Prevention and Treatment (Peking University), Ministry of Education, Beijing 100034, China

**Keywords:** digital health interventions, chronic kidney disease, primary care, systematic review, meta-analysis

## Abstract

Background: Chronic kidney disease (CKD) is a significant public health issue globally. The importance of its timely identification and early intervention is paramount. However, a systematic approach for early CKD management in the primary care setting is currently lacking, receiving less attention compared to upstream risk factors such as diabetes and hypertension. This oversight may lead to a failure in meeting quality-of-care indicators. Digital health interventions (DHIs), which leverage digital tools to enhance healthcare delivery, have shown effectiveness in managing chronic diseases and improving the quality, safety, and efficiency of primary care. Our research aimed to evaluate the effectiveness of DHIs in the care process, focusing on their reach, uptake, and feasibility. Methods: In this systematic review and meta-analysis, we searched PubMed, Embase, Cochrane Central Register of Controlled Trials, Web of Science, and ClinicalTrials.gov for randomized controlled trials (RCTs) assessing DHIs’ effectiveness in CKD patient care among adults in primary care settings. The search, conducted on 30 June 2023, included studies published in English from 1 January 2009. Screening was conducted using Covidence, adhering to Cochrane’s guidelines for data extraction. We primarily evaluated changes in care processes (testing, documentation, medication use, etc.) and the use of renin–angiotensin–aldosterone system inhibitors (RAASi), referrals, among others. Multilevel meta-analysis was employed to address within-study clustering, and meta-regression analyzed the impact of study characteristics on heterogeneity in effect sizes. Clinical endpoints were recorded where available. Bias risk was assessed using the Cochrane Risk of Bias 2 tool. Data on reach, uptake, and feasibility were narratively summarized. The study is registered with PROSPERO (CRD42023449098). Results: From 679 records, 12 RCTs were included in the narrative synthesis, and 6 studies (encompassing 7 trials) in the meta-analysis. The trials indicated a −0.85% change (95%CI, −5.82% to 4.11%) in the proportion of patients receiving desired care. This result showed considerable heterogeneity (I2 = 91.9%). One study characteristic (co-intervention, education) correlated with larger effects. Although including co-intervention in multivariable meta-regression was significant, it did not diminish heterogeneity. The reported reach varied and was not high, while the uptake was relatively high. Most studies did not explicitly address feasibility, though some statements implied its evaluation. Conclusions: The current literature on the impact of DHIs in community-based CKD care is limited. The studies suggest a non-significant effect of DHIs on enhancing CKD management in community settings, marked by significant heterogeneity. Future research should focus on rigorous, methodologically sound implementations to better assess the effectiveness of DHIs in the primary care management of CKD.

## 1. Introduction

Chronic kidney disease (CKD) is a leading public health problem worldwide. In 2017, the Global Burden Disease (GBD) project identified 697.5 million cases of all-stage CKD, with a prevalence of 9.1% (8.5–9.8). CKD has become a prominent noncommunicable cause of mortality worldwide [1]. By 2022, a joint statement indicated that the current number of individuals affected by CKD was estimated to be 843.6 million [2], approximately double the number of people living with diabetes (422 million) [3].

The significance of timely identification and early intervention in CKD cannot be overstated, as the advancement of CKD is associated with devastating clinical consequences such as end-stage kidney disease (ESKD), cardiovascular outcome, and increased death rates [4]. However, at present, there is no accepted systematic approach for the early management of CKD in primary care settings. CKD is usually insidious, with most affected individuals remaining asymptomatic until the disease becomes advanced. Furthermore, patients at high risk of disease development have a limited time window for therapeutic intervention before being referred to nephrology. This is because certain disease-modifying medications can only delay the progression of the disease, rather than prevent it entirely, consequently leading to higher rates of complications and mortality.

Moreover, kidney health receives significantly less focus compared to primary upstream risk factors such as diabetes and hypertension, both from public health authorities and the general population [5,6]. Primary care providers may be under-aware of current CKD treatment guidelines, potentially leading to a failure to meet quality-of-care indicators [7]. 

The primary healthcare system serves as a health gatekeeper and provides a foundation for policy and practice improvements to ensure the efficient delivery of high-quality primary healthcare. However, the focus on primary prevention and management for CKD remains suboptimal, especially in screening and care processes [8,9]. 

Digital health interventions (DHIs) [10] are technologies centered on delivering health services and information through digital and communication tools. They include a wide range of applications such as mobile health apps, wearable devices, telehealth or telemedicine services, and e-prescriptions. These interventions aim to improve patient care, support health professionals, enhance public health services, and optimize health system management. Their scope ranges from providing direct patient care and support to managing data and resources in healthcare settings. The widespread use of electronic health records (EHRs) in recent decades has led to the delivery of DHIs through information technology to improve the quality, safety, and efficiency of healthcare, particularly in primary care [10]. 

Previous reviews [11] have assessed the efficacy of digital health in the management of CKD. However, these reviews have not specifically addressed the impact on improving the process of care for CKD within a community setting, nor have they examined implementation indicators such as reach, uptake, and feasibility at the practical level.

To the best of our knowledge, this systematic review and meta-analysis is the first to synthesize the evidence on the effectiveness of DHIs for managing CKD in primary care settings. Our research aimed to evaluate the effectiveness of DHIs in the process of care and report on their reach, uptake, and feasibility.

## 2. Methods

### 2.1. Search Strategy and Selection Criteria 

Our systematic review protocol, registered in PROSPERO (CRD42023449098), follows the 2020 PRISMA guidelines [12]. We conducted searches on PubMed, Embase, Cochrane Central Register of Controlled Trials, Web of Science, and ClinicalTrials.gov on 30 June 2023, using MeSH terms and broad search terms. We also manually searched the reference lists of related reviews on digital health interventions. The complete search is detailed in Supplementary Appendix S1.

Duplicates among the retrieved citations were removed using EndNote 20 (Thomson ResearchSoft, Stamford, CT, USA). The remaining full articles were then imported into Covidence systematic review software (Veritas Health Innovation, Melbourne, Australia). Two independent researchers (D.Z. and L.Y.) screened the titles and abstracts of the identified articles based on the predefined strategies. The full inclusion and exclusion criteria are listed in Supplementary Table S1. We considered randomized controlled trials (RCTs), cluster RCTs, pilot RCTs, and prospective RCTs conducted in adults within primary care settings that implemented digital health interventions (e.g., screening, diagnostic, medication adherence, and clinical outcome) and published in English from 1 January 2009 to 30 June 2023. Studies involving permanent dialysis treatment were excluded. Trials with control groups receiving substantial digital tool interventions were also excluded. Considering the relatively recent widespread adoption of digital health applications, the start date was set to 2009 to reflect contemporary practice [13]. The full texts of potentially relevant articles were independently assessed by the two reviewers. Discrepancies were resolved by arbitration from a third reviewer. 

### 2.2. Outcomes

The Reach, Effectiveness, Adoption, Implementation, and Maintenance (RE-AIM) model framework guided our evaluation of implementation research [14]. We defined adoption or uptake as the reported action of using the intervention or health promotion program [14,15]. Our focus was primarily on the effectiveness in processes of care, such as testing, documentation, and medication use (e.g., renin–angiotensin–aldosterone system inhibitors (RAASi), referrals). However, we also recorded clinical outcomes, including intermediate endpoints like serum creatinine and blood pressure, when reported. 

### 2.3. Data Extraction

The data extraction form, based on the Cochrane Handbook for Systematic Reviews of Interventions [16], was completed using Covidence systematic review software. For eligible articles, two reviewers independently extracted details including publication data, author information (names, affiliations, funding, and conflicts of interest), study characteristics (start and end date, country, design, purpose, blinding and randomization method, retention rate, and statistical analyses), participant demographics (condition of kidney disease, severity of disease, comorbidities, inclusion and exclusion criteria, sample size, recruitment process, and demographics), intervention (type, duration, frequency, and primary and secondary outcomes) and comparison, results (timepoint for follow-up, and primary and secondary outcomes), and conclusions. 

### 2.4. Quality Assessment

The risk of bias in the included studies was independently assessed by two reviewers using the Cochrane risk-of-bias version 2 tool. Considerations included the randomization process, deviations from intended interventions, missing outcome data, the measurement of outcomes, and the selection of reported result. We used the pertinent versions of the tool to appraise the quality in included RCTs and cluster RCTs. Discrepancies were resolved through discussion and consensus.

### 2.5. Evaluation of Reach, Uptake, and Feasibility of Interventions

Data on reach, uptake, and feasibility were extracted from studies reporting relevant information. Reach was defined as the intended audience’s contact with the intervention [17]. Feasibility encompassed acceptability, adherence, cost-effectiveness likelihood, and provider capacity to deliver the intervention [18]. Based on the Reach, Effectiveness, Adoption, Implementation, and Maintenance (RE-AIM) framework, adoption or uptake was defined as the reported action of taking up or making use of the intervention or health promotion program [14]. We considered reach and uptake at the individual intervention participant level.

### 2.6. Data Analysis

We used a multilevel meta-analysis to estimate absolute improvements in care processes between intervention and control groups, accounting for study heterogeneity and the clustering of multiple outcomes from the same patients. Clustered designs, which means assigning intervention levels to the provider group rather than to the individual patient, did not always report cluster-adjusted estimates. Our study considered clustering through multiplying the standard error of risk differences by the square root of the design effect [19]. For studies without reported intraclass correlation coefficients (ICCs), ICCs were imputed from a published database [20]. We calculated the median ICCs for process measures across the 139 studies in this database and applied the relevant value to a given study.

Univariate and multivariable meta-regression analyses were conducted to examine effects based on study characteristics. These analyses estimated the difference in absolute improvements reported between studies with and without each intervention. Furthermore, a meta-regression model was fitted, including covariates with a *p* value less than 0.1 from the univariate analyses. This was done to identify study and intervention features that predicted larger effects and to determine if heterogeneity could be reduced. We used the I^2^ statistic to summarize statistical heterogeneity. With regard to clinical endpoints, we performed a meta-analysis of changes in systolic blood pressure, which was the most commonly reported continuous clinical endpoint.

All statistical analyses were conducted using R Software, version 4.2.2 (R Foundation for Statistical Computing, Vienna, Australia). The *Rma.mv* function from the “metafor” library was utilized to fit all multilevel models.

## 3. Results

**Figure 1:**
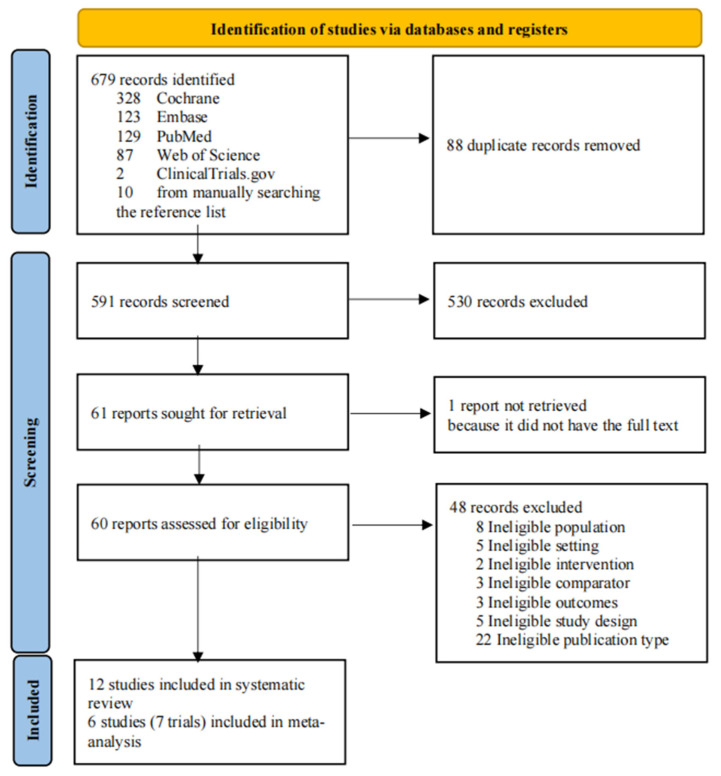
Flow of studies through the review process.

This study identified 679 records, comprising 669 from the database searches and 10 manually sourced from relevant systematic review citations. After eliminating 88 duplicates, title and abstract screening led to the exclusion of 591 records. Of the 61 records subjected to full-text examination, 1 was unavailable in full text. Screening the full text of 60 articles resulted in 48 exclusions, leaving 12 for inclusion in the review. These comprised six studies (encompassing seven trials) reporting on process of care outcome indicators (Figure 1).

The 12 studies primarily originated from the USA (n = 8), followed by 3 in Europe and 1 in Bangladesh. A majority (10 studies) were conducted from 2013 onwards, with the remaining 2 between 2009 and 2012. All studies utilized RCT designs, including one parallel, three cluster, and two 2 × 2 factorial designs. Interventions focused on patients in six studies, on providers in five, and on both in one study. Detailed information regarding the studies included in the analysis is presented in Supplementary Table S2.

**Figure 2:**
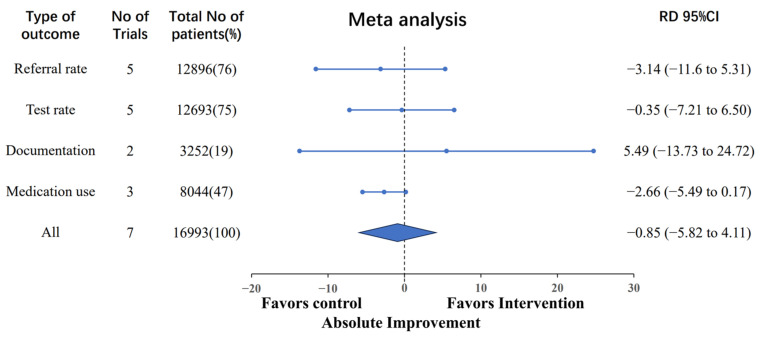
Absolute changes in process of care by different categories of clinical care. Note. The “Referral rate” refers to the frequency of nephrology referrals. The “Test rate” encompasses assessments such as proteinuria evaluation, eGFR testing, hemoglobin measurement, serum phosphorus analysis, 25-Hydroxy vitamin D assessment, and parathyroid hormone measurement. The “Documentation” pertains to the recording of CKD information within the EMR. The “Medication use” signifies the prescription and utilization of ACE inhibitors/ARBs and statins.

Employing a multilevel meta-analysis model (Figure 2), we analyzed incremental improvements in the literature. The findings indicated that DHIs led to a non-significant overall absolute improvement of −0.85% (95% CI, −5.82% to 4.11%) in optimal process of care among patients compared to control groups. In terms of specific process of care characteristics, the referral rate showed a −3.14% difference (95% CI, −11.60% to 5.31%), the testing rates improved by 0.35% (95% CI, −7.21% to 6.50%), the CKD documentation by 5.49% (95% CI, −13.73% to 24.72%), and the medication use decreased by −2.66% (95% CI, −5.49% to 0.17%). Supplementary Figure S2 details models for each category.

The univariate meta-regression analyses (Table 1) revealed a 4.58% improvement (95% CI, −0.71% to 9.87%) in processes of care when education was a co-intervention, compared to a −4.69% change (95% CI, −9.42% to 0.04%) in studies without this aspect, a statistically significant difference (*p* = 0.01).

**Figure 3:**
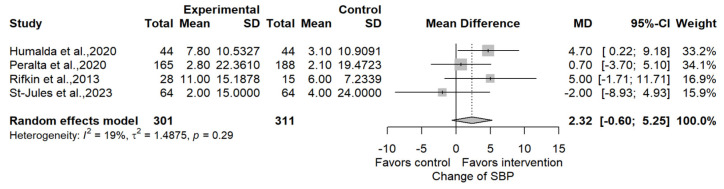
Change in systolic blood pressure [21,22,23,24].

The multivariable regression analysis (Table 2), including co-intervention and follow-up time (both with *p* < 0.1 in the univariate regression), identified co-intervention (*p* = 0.03) as a significantly impacting process of care change. However, high heterogeneity persisted; the initial I^2^ was 91.9% and remained high at I_2_ = 87.3% even after accounting for co-intervention and follow-up time. Systolic blood pressure, the most reported continuous clinical endpoint [21,22,23,24], changed insignificantly by 2.32 mmHg (95%CI, −0.60 to 5.25) (Figure 3).

Sensitivity analyses using odds ratios instead of risk differences showed an overall odds ratio of 0.98 (95%CI, 0.77 to 1.25), underscoring the stability and robustness of conclusions when using odds ratios [25]. 

Regarding reach, uptake, and feasibility, six studies reported intervention reach with a median of 24.6% (range: 6.2–45), while four reported a median of 50% (range: 31.5–63.8) assigned to intervention groups (two studies did not report applicable reach) (Table 3). Intervention uptake, reported in five studies, had a median of 74% (range: 41–100). Although eight studies did not explicitly deem their interventions feasible, they reported effectiveness, reliability, high satisfaction, research participation willingness, and adherence. The authors’ interpretations of feasibility varied among studies, reflecting differences in feasibility measurement components. 

## 4. Discussion

This systematic review synthesized evidence from 12 studies on the effectiveness, reach, uptake, and feasibility of DHIs in managing the care process of 23,945 patients with CKD in community settings. The meta-analysis of seven trials indicated no statistically significant difference in overall and specific care process elements compared to control groups. The reported reach was relatively low, and data on uptake were scarce.

Co-intervention (education) showed statistical significance in both the univariate and multivariate regression analyses. However, this factor did not fully account for the observed heterogeneity across studies. The overall heterogeneity was high at 91.9%, remaining substantial at 87.3% even after the multivariate regression. This suggests the presence of potential wide and non-random variations, the specific reasons for which are currently unknown.

A similar high level of heterogeneity was noted in other systematic reviews. Siopis et al. and Kwan et al. [34,35], who examined the impact of a clinical decision support system (CDSS) on desired care, reported substantial heterogeneity (I^2^ = 76% to 89%), with subgroup analyses unable to clarify the underlying causes. Other reviews [11,36] also failed to identify the origins of significant variations through subgroup analyses.

Several factors might explain the limited efficacy of DHIs. First, some studies [26,30] indicate that the intervention type employed is passive alert, wherein users can only access alert information by clicking on the relevant page, leading to the less frequent clicks and negative pronounced intervention effect. Conversely, pop-up alerts might cause user fatigue, impeding workflow integration. Second, the limited sample size and the patients of early stage CKD may have resulted in too small a number of patients progressing to the advanced stage, hence yielding no significant differences during the intervention monitoring period [22]. Therefore, it is recommended that future research should be undertaken to address the issues of sample size. Third, studies with patients mostly in CKD stages 3–5 [30] showed that referrals often focused on comorbidities rather than nephrology, suggesting a need to consider potential attrition when considering referrals. Fourth, two studies [29,32] indicated limited potential for enhancing DHI management due to high initial examination and medication levels in populations affiliated with university hospitals or general clinics with extensive eGFR implementation. Fifth, the limited CKD knowledge among users, especially primary care providers (PCPs), might lead to an underestimation of alerts and impact medical decision making [35]. Consequently, this can impact the communication between PCPs and patients during medical decision-making processes. Additionally, the active involvement of patients in the intervention process of a DHI is often contingent upon their knowledge of CKD management. As mentioned before, the meta-regression analysis identified potential predictors of Co-intervention (education), which aligns with the findings of a previous systematic review [35] emphasizing the significance of educating users during DHIs.

Lastly, within the community context, the efficacy of the DHIs is limited by various external circumstances. For instance, community-based DHIs face challenges like early CKD diagnosis and treatment awareness, adherence to guidelines, and time constraints for patient education [37,38].

The findings align with other scholarly sources. Stevenson et al. [11] and Galbraith et al. [39] found diverse eHealth intervention concepts and technologies, leading to insufficient evidence for efficacy recommendations. Kwan et al. [35] reported a modest increase in desired care element receipt due to CDSS, with education emerging as a significant predictive factor.

For future research and practical application, it is recommended to integrate alert systems effectively with user workflows. Second, future study should avoid restricting study populations to those with higher baseline levels. Third, when implementing the DHIs, it is crucial to prioritize the education of primary care providers (PCPs) and patients on the management of CKD. Fourth, further investigation should examine and resolve potential obstacles to the implementation of DHIs in a community-based context.

The limitations of the included studies must be acknowledged. First, there is significant heterogeneity among the studies, with differences in intervention types, objectives, and target populations. Second, most studies focused on short-term clinical parameters such as blood pressure and serum creatinine rather than longer term outcomes like hospitalization and mortality, due to the short duration of the RCTs. Third, the assessment of reach, uptake, and feasibility is not comprehensive, with future research needed to explore these aspects more thoroughly and develop robust evaluation frameworks. Additionally, it is important to acknowledge that the limited number of studies incorporated in the meta-analysis and meta-regression within this review may impact the power of the data analysis findings. Therefore, it is advisable to interpret the conclusions of this study with caution.

In conclusion, the current literature on DHIs in community-based CKD care suggests limited efficacy and considerable heterogeneity. Future research should focus on methodologically sound implementations to assess the effectiveness of DHIs in primary care CKD management more rigorously.

In terms of the risk of bias of the studies, three studies were classified at high risk of bias, five at moderate risk, and four at low risk (Supplementary Figure S1). Two studies [24,30] were high-risk due to unblinded intervention allocation, and one study [28] was high-risk due to unblinded outcome measurement. Four studies had moderate risk due to potential randomization process issues, and four either lacked or did not mention blinding in the allocation process.

## 5. Conclusions

The literature on the impact of DHIs in community-based CKD care is limited. Existing studies indicate that the efficacy of DHIs in enhancing CKD management is not significant and exhibits considerable heterogeneity. Future research should undertake rigorous and methodologically sound investigations to examine DHIs’ effectiveness in CKD management within primary care more effectively.

## Figures and Tables

**Table 1:** Absolute changes in process of care by study feature.

Study Feature		Correlation Coefficient	Effect Size (95% Confidence Interval)	*p* Value
Year		−0.2781		0.74
Number of patients		0.0015		0.22
Follow-up time		−0.7280		0.09
Area	USA		−0.89 (−6.89, 5.10)	0.99
	Europe		−0.93 (−14.62, 12.75)	
Age †	Young		−3.64 (−10.94, 3.65)	0.30
	Old		1.46 (−5.16, 8.07)	
Gender	Male		−4.60 (−19.70, 10.51)	0.60
	Female		−0.37 (−5.95, 5.21)	
Setting	Community		−1.59 (−11.46, 78.27)	0.86
	Others		−0.59 (−7.10, 5.92)	
Intervention type	CDSS		1.59 (−3.74, 6.92)	0.11
	Other		−6.10 (−14.08, 1.88)	
Co-intervention (education)	Yes		4.58 (−0.71, 9.87)	0.01 *
	No		−4.69 (−9.42, 0.04)	

CDSS: clinical decision support system. † Trials with a mean age of participants higher than the median mean age of all included trials were categorized as “Old”, whereas those with a lower mean age were categorized as “Young”. * *p* value < 0.05.

**Table 2:** Multivariable meta-regression model for absolute changes in process of care by study feature.

Study Feature	*p* Value
Co-intervention (education)	0.03 *
Follow time	0.15

* *p* value < 0.05.

**Table 3:** Reach, uptake, and feasibility of the included studies.

	Reach	Uptake	Feasibility
Abdel-Kader [26] 2011	32.6% (50%)	NR	NR, reported high recruitment
Bhardwaja [27] 2011	NR (50.4%)	100%	reported effectiveness and reliability
Blakeman [28] 2014	33.8% (48.9%)	telephone 67.8%, web 30.7%	reported acceptability and cost-effectiveness
Humalda [21] 2020	NR (52.5%)	88%	NR
Navaneethan [29] 2017	NA	NR	NR
Peralta [22] 2020	16.6% (31.5%)	74%	NR, reported low opt-out rate by physicians and patients demonstrating willingness to participate in research
Rifkin [23] 2013	8.9% (63.8%)	NR	reported high willingness to continue using the device
Samal [30] 2022	NR (50.0%)	41.0%	NR
Sarker [31] 2022	45.0% (50.0%)	NR	NR
Sequist [32] 2018	NR (50.9%)	NR	NR, reported high satisfaction
St-Jules [24] 2023	NA	NR	NR, reported high adherence
van Gelder [33] 2017	6.2% (42.5%)	NR	NR, reported high satisfaction

NR: Not reported. NA: Not applicable.

## Data Availability

The original contributions presented in the study are included in the article and Supplementary Material, further inquiries can be directed to the corresponding authors.

## Supplementary Materials

The following supporting information can be downloaded at: https://www.mdpi.com/article/10.3390/jcm13020364/s1**.**
