# Supplementary material for: Digital Health Interventions for Quality Improvements in Chronic Kidney Disease Primary Care: A Systematic Review and Meta-Analysis of Randomized Controlled Trials
Source: J Clin Med. 2024 Jan 9;13(2):364. doi: 10.3390/jcm13020364 (PMC10816029; doi:10.3390/jcm13020364)

Supplementary Appendix

Supplementary Appendix S1. Search Strings....2
Supplementary Appendix S2. Reference List of Included Studies....10
Supplementary Table S1. Eligibility Criteria for Study Inclusion....11
Supplementary Table S2. Characteristics of Included Studies....12
Supplementary Figure S1. Risk of Bias Report of Included Studies....17
Supplrmentary Figure S2a. Abosolute Improvements in Referral Rate.....18
Supplementary Figure S2b. Abosolute Improvements in Test Rate....18
Supplementary Figure S2c. Abosolute Improvements in CKD Documentation....19
Supplementary Figure S2d. Abosolute Improvements in Medication Use....19
Supplementary Figure S2e. Absolute Improvement of processes of care....20

Supplementary Appendix S1. Search Strings

PubMed

Data of search: 30/6/2023

#	Search	Results
1	"Renal Insufficiency, Chronic"[Mesh]	134,097
2	"Chronic Renal Insufficiencies"[TIAB] OR "Renal Insufficiencies, Chronic"[TIAB] OR "(Chronic Renal Insufficiency"[TIAB] OR "Kidney Insufficiency, Chronic"[TIAB] OR "Chronic Kidney Insufficiency"[TIAB] OR "Chronic Kidney Insufficiencies"[TIAB] OR "Kidney Insufficiencies, Chronic"[TIAB] OR "Chronic Kidney Diseases"[TIAB] OR "Chronic Kidney Disease"[TIAB] OR "Disease, Chronic Kidney"[TIAB] OR "Diseases, Chronic Kidney"[TIAB] OR "Kidney Disease, Chronic"[TIAB] OR "Kidney Diseases, Chronic"[TIAB] OR "Chronic Renal Diseases"[TIAB] OR "Chronic Renal Disease"[TIAB] OR "Disease, Chronic Renal"[TIAB] OR "Diseases, Chronic Renal"[TIAB] OR "Renal Disease, Chronic"[TIAB] OR "Renal Diseases, Chronic"[TIAB]	82,156
3	CKD[TIAB] OR CRD[TIAB] OR"kidney disease*"[tiab] OR "renal disease*"[tiab]	186,167
4	"Diabetic Nephropathies"[Mesh]	29,441
5	"Nephropathies, Diabetic"[TIAB] OR "Nephropathy, Diabetic"[TIAB] OR "Diabetic Nephropathy"[TIAB] OR "Diabetic Kidney Disease"[TIAB] OR "Diabetic Kidney Diseases"[TIAB] OR "Kidney Disease, Diabetic"[TIAB] OR "Kidney Diseases, Diabetic"[TIAB] OR "Diabetic Glomerulosclerosis"[TIAB] OR "Glomerulosclerosis, Diabetic"[TIAB] OR "Intracapillary Glomerulosclerosis"[TIAB] OR "Nodular Glomerulosclerosis"[TIAB] OR "Glomerulosclerosis, Nodular"[TIAB] OR "Kimmelstiel-Wilson Syndrome"[TIAB] OR "Kimmelstiel Wilson Syndrome"[TIAB] OR "Syndrome, Kimmelstiel-Wilson"[TIAB] OR "Kimmelstiel-Wilson Disease"[TIAB] OR "Kimmelstiel Wilson Disease"[TIAB]	27,049
6	"Hypertension, Renal"[Mesh] OR "Hypertensions, Renal"[TIAB] OR "Renal Hypertension"[TIAB] OR "Renal Hypertensions"[TIAB]	20,135
7	"Hypertension, Renal"[Mesh] OR "Renovascular Hypertension"[TIAB] OR "Hypertension, Goldblatt"[TIAB] OR "Goldblatt Hypertension"[TIAB] OR "Goldblatt Syndrome"[TIAB] OR "Syndrome, Goldblatt"[TIAB]	20,818
8	#1 OR #2 OR #3 OR #4 OR #5 OR #6 OR #7	297,435
9	"Primary Health Care"[Mesh] OR "Care, Primary Health"[TIAB] OR "Health Care, Primary"[TIAB] OR "Primary Healthcare"[TIAB] OR "Healthcare, Primary"[TIAB] OR "Primary Care"[TIAB] OR "Care, Primary"[TIAB]	280,843
10	"Physicians, Primary Care"[Mesh] OR "Physician, Primary Care"[TIAB] OR "Primary Care Physician"[TIAB] OR "Primary Care Physicians"[TIAB]	24,713
11	"Family Practice"[Mesh] OR "Family Practices"[TIAB] OR "Practice, Family"[TIAB] OR "Practices, Family"[TIAB]	68,339
12	"General Practice"[Mesh] OR "Practice, General"[TIAB]	78,733
13	"Community Health Services"[Mesh] OR "Community Health Service"[TIAB] OR "Health Service, Community"[TIAB] OR "Service, Community Health"[TIAB] OR "Services, Community Health"[TIAB]	334,524

	OR "Health Services, Community"[TIAB] OR "Community Health Care"[TIAB] OR "Health Care, Community"[TIAB] OR "Community Healthcare"[TIAB] OR "Healthcare, Community"[TIAB]	
14	"Geriatrics"[Mesh] OR "Gerontology"[TIAB]	34,339
15	"Internal Medicine"[Mesh] OR "Medicine, Internal"[TIAB] OR "General Internal Medicine"[TIAB] OR "Internal Medicine, General"[TIAB] OR "Medicine, General Internal"[TIAB]	118,434
16	#9 OR #10 OR #11 OR #12 OR #13 OR #14 OR #15	787,858
17	#8 AND #16	8,994
18	"Telemedicine"[Mesh]	44,647
19	"Tele-Referral"[TIAB] OR "Tele Referral"[TIAB] OR "Tele-Referrals"[TIAB] OR "Virtual Medicine"[TIAB] OR "Medicine, Virtual"[TIAB] OR "Tele-Intensive Care"[TIAB] OR "Tele Intensive Care"[TIAB] OR "Tele-ICU"[TIAB] OR "Tele ICU"[TIAB] OR "Mobile Health"[TIAB] OR "Health, Mobile"[TIAB] OR "mHealth"[TIAB] OR "Telehealth"[TIAB] OR "eHealth"[TIAB]	35,044
20	"digital health"[TIAB] OR "digital health intervention"[TIAB] OR "digital health technology"[TIAB]	7,583
21	"decision support systems, clinical"[MeSH] OR "Clinical Decision Support Systems"[TIAB] OR "Clinical Decision Support System"[TIAB] OR "Clinical Decision Support"[TIAB] OR "Clinical Decision Supports"[TIAB] OR "Decision Supports, Clinical"[TIAB] OR "Support, Clinical Decision"[TIAB] OR "Supports, Clinical Decision"[TIAB] OR "Decision Support, Clinical"[TIAB]	14,505
22	"Artificial Intelligence"[Mesh] OR "Intelligence, Artificial"[TIAB] OR "Computational Intelligence"[TIAB] OR "Intelligence, Computational"[TIAB] OR "Machine Intelligence"[TIAB] OR "Intelligence, Machine"[TIAB] OR "Computer Reasoning"[TIAB] OR "Reasoning, Computer"[TIAB] OR "AI (Artificial Intelligence)"[TIAB] OR "Computer Vision Systems"[TIAB] OR "Computer Vision System"[TIAB] OR "System, Computer Vision"[TIAB] OR "Systems, Computer Vision"[TIAB] OR "Vision System, Computer"[TIAB] OR "Vision Systems, Computer"[TIAB] OR "Knowledge Acquisition (Computer)"[TIAB] OR "Acquisition, Knowledge (Computer)"[TIAB] OR "Knowledge Representation (Computer)"[TIAB] OR "Knowledge Representations (Computer)"[TIAB] OR "Representation, Knowledge (Computer)"[TIAB]	174,894
23	"digital health"[TIAB] OR "online health"[TIAB] OR "weight loss app*"[TIAB] OR "weight reduction app*"[TIAB] OR "weight management app*"[TIAB] OR "lipid monitoring app*"[TIAB] OR "lipid management app*"[TIAB] OR "glucose monitoring app*"[TIAB] OR "glucose management app*"[TIAB] OR "health monitoring app*"[TIAB] OR "lifestyle app*"[TIAB] OR "diabetes management app*"[TIAB] OR "hypertension management app*"[TIAB] OR "blood pressure management app*"[TIAB] OR "blood pressure monitoring app*"[TIAB]	10,547
24	"Electronic Health Records"[MeSH] OR "electronic health record*"[tiab] OR "personal health record*"[tiab] OR "electronic medical record*"[tiab] OR "EMR"[tiab] OR "EHR"[tiab] OR "Health Records, Personal"[MeSH] OR "Computer Security"[Mesh] OR "data security"[tiab] OR "cybersecurity"[tiab] OR "cyber security"[tiab] OR "data protect*"[tiab] OR "data encrypt*"[tiab] OR "Cloud Computing"[Mesh] OR "cloud computing"[tiab] OR "cloud process*"[tiab] OR "cognitive comput*"[tiab] OR "Patient Portals"[Mesh] OR "patient web portal*"[tiab] OR "patient web-	565,280

	portal*"[tiab] OR "patient portal*"[tiab] OR "web portal*"[tiab] OR "Health Information Exchange"[MeSH] OR "health information exchange*"[tiab] OR "electronic health information"[tiab] OR "electronic health communication*"[tiab] OR "health information interoperability"[MeSH] OR "interoperability" OR "patient monitor*"[tiab] OR "wearables"[tiab] OR "activity monitor*"[tiab] OR "sensor*"[tiab] OR "physiologic monitoring"[MeSH] OR "physiologic monitoring"[tiab]	
25	"Machine Learning"[MeSH] OR "machine learning"[tiab] OR "machine-learning"[tiab] OR "natural language processing"[tiab] OR "neural network*"[tiab] OR "quantified self"[tiab] OR "connected health"[tiab] OR "big data"[tiab] OR "gamification"[tiab] OR "social media"[tiab] OR "health 2.0"[tiab] OR "personalized genomics"[tiab] OR "precision medicine"[tiab] OR "precision medicine"[MeSH] OR "individualized medicine"[tiab] OR "internet of things"[tiab] OR "IoT"[tiab] OR "IOT"[tiab] OR (("social program*"[tiab] OR "care manage*"[tiab] OR "coordinate* care"[tiab] OR "health benefit*"[tiab] OR insur*[tiab]) AND "digital"[tiab])	279,874
26	#18 OR #19 OR #20 OR #21 OR #22 OR #23 OR #24 OR #25	959,194
27	#17 AND #26	528
28	#17 AND #26	257
29	#17 AND #26	252
30	#17 AND #26	244
31	"Clinical Trial" [Publication Type] OR "Clinical Trials as Topic"[Mesh] OR "Controlled Clinical Trial" [Publication Type] OR "Pragmatic Clinical Trial" [Publication Type] OR "Clinical Trial, Phase IV" [Publication Type] OR "Clinical Trial, Phase III" [Publication Type] OR "Clinical Trial, Phase II" [Publication Type] OR "Clinical Trial, Phase I" [Publication Type] OR "Clinical Trial Protocols as Topic"[Mesh] OR "Clinical Trial Protocol" [Publication Type] OR "Adaptive Clinical Trial" [Publication Type]	1,276,642
32	clinical[tiab] or trial*[tiab] or random*[tiab]	5,721,841
33	#31 or #32	6,110,555
34	#30 and #33	129

Embase

Data of search: 30/6/2023

#	Search	Results
1	'chronic kidney failure'/exp	202284
2	'chronic kidney disease':ti,ab,kw OR 'chronic kidney disorder':ti,ab,kw OR 'chronic kidney insufficiency':ti,ab,kw OR 'chronic nephropathy':ti,ab,kw OR 'chronic renal disease':ti,ab,kw OR 'chronic renal failure':ti,ab,kw OR 'chronic renal insufficiency':ti,ab,kw OR 'kidney chronic failure':ti,ab,kw OR 'kidney disease, chronic':ti,ab,kw OR 'kidney failure, chronic':ti,ab,kw OR 'idney function, chronic disease':ti,ab,kw OR 'renal insufficiency, chronic':ti,ab,kw OR 'chronic kidney failure':ti,ab,kw	164239
3	ckd:ti,ab,kw OR crd:ti,ab,kw	90336
4	'diabetic nephropathy'/exp	54695
5	'diabetes nephropathy':ti,ab,kw OR 'diabetic glomerulopathy':ti,ab,kw OR	40485

	'diabetic glomerulosclerosis':ti,ab,kw OR 'diabetic intercapillary glomerulosclerosis':ti,ab,kw OR 'diabetic kidney disease':ti,ab,kw OR 'diabetic nephropathies':ti,ab,kw OR 'diabetic nephrosclerosis':ti,ab,kw OR 'glomerulonecrosis, intercapillary':ti,ab,kw OR 'glomerulosclerosis, diabetic':ti,ab,kw OR 'glomerulosclerosis, intercapillary':ti,ab,kw OR 'intercapillary glomerulosclerosis':ti,ab,kw OR 'kimmelstiehl wilson syndrome':ti,ab,kw OR 'kimmelstiel wilson disease':ti,ab,kw OR 'kimmelstiel wilson nephropathy':ti,ab,kw OR 'kimmelstiel wilson syndrome':ti,ab,kw OR 'nephropathy, diabetic':ti,ab,kw OR 'diabetic nephropathy':ti,ab,kw	
6	'renovascular hypertension'/exp OR 'goldblatt hypertension':ti,ab,kw OR 'goldblatt kidney':ti,ab,kw OR 'hypertension, goldblatt':ti,ab,kw OR 'hypertension, renal':ti,ab,kw OR 'hypertension, renovascular':ti,ab,kw OR 'juxtaglomerular hypertension':ti,ab,kw OR 'kidney hypertension':ti,ab,kw OR 'kidney vascular hypertension':ti,ab,kw OR 'nephrogenic hypertension':ti,ab,kw OR 'nephrogenous hypertension':ti,ab,kw OR 'renal hypertension':ti,ab,kw OR 'renal vascular hypertension':ti,ab,kw OR 'renoarterial hypertension':ti,ab,kw OR 'renovascular hypertension':ti,ab,kw	27810
7	#1 OR #2 OR #3 OR #4 OR #5 OR #6	335969
8	'primary health care'/exp	206278
9	'first line care':ti,ab,kw OR 'health care, primary':ti,ab,kw OR 'primary care nursing':ti,ab,kw OR 'primary healthcare':ti,ab,kw OR 'primary nursing care':ti,ab,kw OR 'primary health care':ti,ab,kw	48255
10	'general practitioner'/exp OR 'family doctor':ti,ab,kw OR 'family physician':ti,ab,kw OR 'family physicians':ti,ab,kw OR 'family practitioner':ti,ab,kw OR 'general physician':ti,ab,kw OR 'general practice physician':ti,ab,kw OR 'general practitioners':ti,ab,kw OR 'gp (general practitioner)':ti,ab,kw OR 'physicians, family':ti,ab,kw OR 'physicians, primary care':ti,ab,kw OR 'practitioner, general':ti,ab,kw OR 'primary care doctor':ti,ab,kw OR 'primary care physician':ti,ab,kw OR 'primary care physicians':ti,ab,kw OR 'general practitioner':ti,ab,kw	181017
11	'general practice'/exp OR 'family practice':ti,ab,kw OR 'general medical practice':ti,ab,kw OR 'general medicine':ti,ab,kw OR 'general practice':ti,ab,kw	120750
12	'primary medical care'/exp OR 'medical care, primary':ti,ab,kw OR 'primary care':ti,ab,kw OR 'primary medical care':ti,ab,kw	229423
13	'community care'/exp OR 'community care service':ti,ab,kw OR 'community health action':ti,ab,kw OR 'community health care':ti,ab,kw OR 'community health service':ti,ab,kw OR 'community health services':ti,ab,kw OR 'community networks':ti,ab,kw OR 'religious missions':ti,ab,kw OR 'uncompensated care':ti,ab,kw OR 'community care':ti,ab,kw	145771
14	'internal medicine'/exp OR 'medicine, internal':ti,ab,kw OR 'internal medicine':ti,ab,kw	354147
15	'geriatrics'/exp OR 'geriatric aspects':ti,ab,kw OR 'geriatric medicine':ti,ab,kw OR 'geriatric practice':ti,ab,kw OR 'geriatry':ti,ab,kw OR 'sociogeriatrics':ti,ab,kw OR 'geriatrics':ti,ab,kw	74031
16	#8 OR #9 OR #10 OR #11 OR #12 OR #13 OR #14 OR #15	1002133
17	'telehealth'/exp	85023
18	'e-health':ti,ab,kw OR 'ehealth':ti,ab,kw OR 'tele-health':ti,ab,kw OR 'telehealth':ti,ab,kw	29380
19	'digital health':ti,ab,kw OR 'digital health intervention':ti,ab,kw OR 'digital	7650

	health technology':ti,ab,kw	
20	'clinical decision support system'/exp OR 'cds system (clinical decision support system)':ti,ab,kw OR 'clinical decision support (cds) system':ti,ab,kw OR 'clinical decision support systems':ti,ab,kw OR 'clinician decision support system':ti,ab,kw OR 'decision support systems, clinical':ti,ab,kw OR 'clinical decision support system':ti,ab,kw	7836
21	'artificial intelligence'/exp OR 'machine intelligence':ti,ab,kw OR 'artificial intelligence':ti,ab,kw	88763
22	'mhealth':ti,ab,kw OR 'mobile health':ti,ab,kw OR 'health app*':ti,ab,kw OR 'digital health':ti,ab,kw OR 'online health':ti,ab,kw OR 'weight loss app*':ti,ab,kw OR 'weight reduction app*':ti,ab,kw OR 'weight management app*':ti,ab,kw OR 'lipid monitoring app*':ti,ab,kw OR 'lipid management app*':ti,ab,kw OR 'glucose monitoring app*':ti,ab,kw OR 'glucose management app*':ti,ab,kw OR 'health monitoring app*':ti,ab,kw OR 'lifestyle app*':ti,ab,kw OR 'diabetes management app*':ti,ab,kw OR 'hypertension management app*':ti,ab,kw OR 'blood pressure management app*':ti,ab,kw OR 'blood pressure monitoring app*':ti,ab,kw	33719
23	#17 OR #18 OR #19 OR #20 OR #21 OR #22	210953
24	#7 AND #16	18166
25	#23 AND #24	365
26	#25 AND ([adult]/lim OR [aged]/lim)	204
27	#25 AND ([adult]/lim OR [aged]/lim) AND [english]/lim	204
28	#27 AND (2010:py OR 2011:py OR 2012:py OR 2013:py OR 2014:py OR 2015:py OR 2016:py OR 2017:py OR 2018:py OR 2019:py OR 2020:py OR 2021:py OR 2022:py OR 2023:py)	202
29	#28 AND ('clinical protocol'/de OR 'clinical trial'/de OR 'clinical trial topic'/de OR 'comparative effectiveness'/de OR 'comparative study'/de OR 'controlled study'/de OR 'intervention study'/de OR 'pragmatic trial'/de OR 'randomized controlled trial'/de OR 'randomized controlled trial topic'/de)	123

Cochrane

Data of search: 30/6/2023

#	Search	Results
1	MeSH descriptor: [Renal Insufficiency, Chronic] explode all trees	8588
2	(Renal Insufficiency, Chronic OR Diseases, Chronic Kidney OR Disease, Chronic Renal OR Disease, Chronic Kidney OR Chronic Renal Disease OR Kidney Disease, Chronic OR Diseases, Chronic Renal OR Chronic Renal Diseases OR Chronic Kidney Disease OR Kidney Diseases, Chronic OR Renal Disease, Chronic OR Renal Diseases, Chronic OR Chronic Kidney Diseases OR Chronic Renal Insufficiency OR Chronic Kidney Insufficiencies OR Kidney Insufficiency, Chronic OR Renal Insufficiencies, Chronic OR Chronic Renal Insufficiencies OR Kidney Insufficiencies, Chronic OR Chronic Kidney Insufficiency):ti,ab,kw	19301
3	(CKD OR CRD):ti,ab,kw	7817
4	MeSH descriptor: [Diabetic Nephropathies] explode all trees	1793
5	(Diabetic Nephropathies OR Diabetic Glomerulosclerosis OR Glomerulosclerosis, Diabetic OR Kimmelstiel-Wilson Syndrome OR Kimmelstiel-Wilson Disease OR Glomerulosclerosis, Nodular OR Nodular Glomerulosclerosis OR Kimmelstiel Wilson Disease OR Intracapillary Glomerulosclerosis OR Kimmelstiel Wilson Syndrome OR Syndrome, Kimmelstiel-Wilson OR Kidney Disease, Diabetic OR Kidney Diseases, Diabetic OR Diabetic Kidney Diseases OR Nephropathy, Diabetic OR Nephropathies, Diabetic OR Diabetic Kidney Disease OR Diabetic	6684

	Nephropathy):ti,ab,kw	
6	#1 OR #2 OR #3 OR #4 OR #5	28046
7	MeSH descriptor: [Telemedicine] explode all trees	4250
8	(Telemedicine OR Virtual Medicine OR Medicine, Virtual OR Tele-Referrals OR Tele-Referral OR Tele Referral OR Health, Mobile OR mHealth OR Mobile Health OR Telehealth OR eHealth OR Tele-ICU OR Tele Intensive Care OR Tele-Intensive Care OR Tele ICU):ti,ab,kw	17314
9	MeSH descriptor: [Decision Support Systems, Clinical] explode all trees	563
10	(Decision Support Systems, Clinical OR Clinical Decision Support Systems OR Clinical Decision Support System OR Clinical Decision Support OR Clinical Decision Supports OR Decision Supports, Clinical OR Support, Clinical Decision OR Supports, Clinical Decision OR Decision Support, Clinical):ti,ab,kw	6176
11	MeSH descriptor: [Artificial Intelligence] explode all trees	2883
12	(Artificial Intelligence OR Machine Intelligence OR Intelligence, Artificial OR Computer Reasoning OR AI (Artificial Intelligence) OR Intelligence, Computational OR Intelligence, Machine OR Reasoning, Computer OR Computational Intelligence OR Knowledge Acquisition (Computer) OR Acquisition, Knowledge (Computer) OR Representation, Knowledge (Computer) OR Knowledge Representation (Computer) OR Knowledge Representations (Computer) OR Vision System, Computer OR Systems, Computer Vision OR Vision Systems, Computer OR Computer Vision System OR Computer Vision Systems OR System, Computer Vision):ti,ab,kw	2360
13	(health app* OR digital health OR online health OR weight loss app* OR weight reduction app* OR weight management app* OR lipid monitoring app* OR lipid management app* OR glucose monitoring app* OR glucose management app* OR health monitoring app* OR lifestyle app* OR diabetes management app* OR hypertension management app* OR blood pressure management app* OR blood pressure monitoring app* OR teledietetics OR telediet* OR smartphone*):ti,ab,kw	144634
14	#7 OR #8 OR #9 OR #10 OR #11 OR #12 OR #13	159557
15	MeSH descriptor: [Primary Health Care] explode all trees	10333
16	(Primary Health Care OR Care, Primary OR Primary Care OR Healthcare, Primary OR Care, Primary Health OR Health Care, Primary OR Primary Healthcare):ti,ab,kw	117852
17	MeSH descriptor: [General Practice] explode all trees	3038
18	MeSH descriptor: [Family Practice] explode all trees	2243
19	MeSH descriptor: [Physicians, Family] explode all trees	510
20	MeSH descriptor: [Community Health Services] explode all trees	18483
21	(Family Practice OR Practice, Family OR Family Practices OR Practices, Family OR General Practice OR Practice, General OR Physicians, Family OR Family Physician OR Physician, Family OR Family Physicians OR Community Health Services OR Community Health Care OR Healthcare, Community OR Health Care, Community OR Community Healthcare OR Community Health Service OR Health Service, Community OR Health Services, Community OR Service, Community Health OR Services, Community Health):ti,ab,kw	46202
22	#15 OR #16 OR #17 OR #18 OR #19 OR #20 OR #21	159479
23	#14 AND #22	48277
24	#6 AND #23	865

25	#6 AND #23 with Publication Year from 2009 to present, in Trials	777
26	(adult* OR elder*):ti,ab,kw	84398 7
27	(Child* OR adolesc* OR teen*):ti,ab,kw	29522 4
28	#26 NOT #27	70005 9
29	#25 AND #28	328

Web of Science

Data of search: 30/6/2023

#	Search	Results
1	TS=(Chronic Renal Disease OR Chronic Renal Diseases OR Chronic Kidney Disease OR Chronic Kidney Diseases OR Chronic Renal Insufficiency OR Chronic Kidney Insufficiencies OR Chronic Renal Insufficiencies OR Chronic Kidney Insufficiency OR CKD OR CRD)	336844
2	TS=(Diabetic Nephropathies OR Diabetic Glomerulosclerosis OR Kimmelstiel-Wilson Syndrome OR Kimmelstiel-Wilson Disease OR Nodular Glomerulosclerosis OR Kimmelstiel Wilson Disease OR Intracapillary Glomerulosclerosis OR Kimmelstiel Wilson Syndrome OR Diabetic Kidney Diseases OR Diabetic Kidney Disease OR Diabetic Nephropathy)	99195
3	#1 OR #2	407650
4	TS=(Telemedicine OR Virtual Medicine OR Tele-Referrals OR Tele-Referral OR Tele Referral OR mHealth OR Mobile Health OR Telehealth OR eHealth OR Tele-ICU OR Tele Intensive Care OR Tele-Intensive Care OR Tele ICU)	203623
5	TS=(Clinical Decision Support Systems OR Clinical Decision Support System OR Clinical Decision Support OR Clinical Decision Supports)	83355
6	TS=(Artificial Intelligence OR Machine Intelligence OR Computer Reasoning OR AI (Artificial Intelligence) OR Computational Intelligence OR Knowledge Acquisition (Computer) OR Knowledge Representation (Computer) OR Knowledge Representations (Computer) OR Computer Vision System OR Computer Vision Systems)	118946 7
7	TS=(health app* OR digital health OR online health OR weight loss app* OR weight reduction app* OR weight management app* OR lipid monitoring app* OR lipid management app* OR glucose monitoring app* OR glucose management app* OR health monitoring app* OR lifestyle app* OR diabetes management app* OR hypertension management app* OR blood pressure management app* OR blood pressure monitoring app* OR teledietetics OR telediet* OR smartphone*)	341311 2
8	#4 OR #5 OR #6 OR #7	468447 5
9	TS=(Primary Health Care OR Primary Care OR Primary Healthcare OR Family Practice OR Family Practices OR General Practice OR Family Physician OR Family Physicians OR Community Health Services OR Community Health Care OR Community Healthcare OR Community Health Service)	170467 1
10	#8 and #9	450799
11	#3 and #10	4917
12	#3 and #10 and 2010 OR 2009 OR 2011 OR 2012 OR 2013 OR 2014 OR	4005

	2015 OR 2016 OR 2017 OR 2018 OR 2019 OR 2020 OR 2021 OR 2022 OR 2023 (Pulication Year)	
13	#3 and #10 and 2010 OR 2009 OR 2011 OR 2012 OR 2013 OR 2014 OR 2015 OR 2016 OR 2017 OR 2018 OR 2019 OR 2020 OR 2021 OR 2022 OR 2023 (Pulication Year) and Clinical Trial (Pulication Type)	215
14	#3 and #10 and 2010 OR 2009 OR 2011 OR 2012 OR 2013 OR 2014 OR 2015 OR 2016 OR 2017 OR 2018 OR 2019 OR 2020 OR 2021 OR 2022 OR 2023 (Pulication Year) and Clinical Trial (Pulication Type) and English (Language)	214
15	TS=(adult* OR elder*)	8664916
16	TS=(Child* OR adolesc* OR teen*)	5800848
17	#15 not #16	6204065
18	#14 and #17	87

ClinicalTrials.gov

Data of search: 30/6/2023

	Search	Results
Condition OR disease	Renal Insufficiency, Chronic OR Diseases, Chronic Kidney OR Disease, Chronic Renal OR Disease, Chronic Kidney OR Chronic Renal Disease OR Kidney Disease, Chronic OR Diseases, Chronic Renal OR Chronic Renal Diseases OR Chronic Kidney Disease OR Kidney Diseases, Chronic OR Renal Disease, Chronic OR Renal Diseases, Chronic OR Chronic Kidney Diseases OR Chronic Renal Insufficiency OR Chronic Kidney Insufficiencies OR Kidney Insufficiency, Chronic OR Renal Insufficiencies, Chronic OR Chronic Renal Insufficiencies OR Kidney Insufficiencies, Chronic OR Chronic Kidney Insufficiency OR CKD OR CRD OR Diabetic Nephropathies OR Diabetic Glomerulosclerosis OR Glomerulosclerosis, Diabetic OR Kimmelstiel-Wilson Syndrome OR Kimmelstiel-Wilson Disease OR Glomerulosclerosis, Nodular OR Nodular Glomerulosclerosis OR Kimmelstiel Wilson Disease OR Intracapillary Glomerulosclerosis OR Kimmelstiel Wilson Syndrome OR Syndrome, Kimmelstiel-Wilson OR Kidney Disease, Diabetic OR Kidney Diseases, Diabetic OR Diabetic Kidney Diseases OR Nephropathy, Diabetic OR Nephropathies, Diabetic OR Diabetic Kidney Disease OR Diabetic Nephropathy	
Intervention/Treatment	Telemedicine OR Virtual Medicine OR Medicine, Virtual OR Tele-Referrals OR Tele-Referral OR Tele Referral OR Health, Mobile OR mHealth OR Mobile Health OR Telehealth OR eHealth OR Tele-ICU OR Tele Intensive Care OR Tele-Intensive Care OR Tele ICU OR Decision Support Systems, Clinical OR Clinical Decision Support Systems OR Clinical Decision Support System OR Clinical Decision Support OR Clinical Decision Supports OR Decision Supports, Clinical OR Support, Clinical	

	Decision OR Supports, Clinical Decision OR Decision Support, Clinical OR Artificial Intelligence OR Machine Intelligence OR Intelligence, Artificial OR Computer Reasoning OR AI (Artificial Intelligence) OR Intelligence, Computational OR Intelligence, Machine OR Reasoning, Computer OR Computational Intelligence OR Knowledge Acquisition (Computer) OR Acquisition, Knowledge (Computer) OR Representation, Knowledge (Computer) OR Knowledge Representation (Computer) OR Knowledge Representations (Computer) OR Vision System, Computer OR Systems, Computer Vision OR Vision Systems, Computer OR Computer Vision System OR Computer Vision Systems OR System, Computer Vision OR health app* OR digital health OR online health OR weight loss app* OR weight reduction app* OR weight management app* OR lipid monitoring app* OR lipid management app* OR glucose monitoring app* OR glucose management app* OR health monitoring app* OR lifestyle app* OR diabetes management app* OR hypertension management app* OR blood pressure management app* OR blood pressure monitoring app* OR teledietetics OR telediet* OR smartphone*	
Age	Adults	
Study type	Interventional	
Study results	With results	
		2

Supplementary Appendix S2. Reference List of Included Studies

1. Abdel-Kader, K., et al., *Automated clinical reminders for primary care providers in the care of CKD: a small cluster-randomized controlled trial.* Am J Kidney Dis, 2011. **58**(6): p. 894-902.
2. Bhardwaja, B., et al., *- Improving Prescribing Safety in Patients with Renal Insufficiency in the Ambulatory Setting: The Drug Renal Alert Pharmacy (DRAP) Program.* 2011. **- 31**(- 4): p. - 356.
3. Blakeman, T., et al., *Effect of information and telephone-guided access to community support for people with chronic kidney disease: randomised controlled trial.* PLoS One, 2014. **9**(10): p. e109135.
4. Humalda, J.K., et al., *A Self-management Approach for Dietary Sodium Restriction in Patients With CKD: a Randomized Controlled Trial.* American journal of kidney diseases, 2020. **75**(6): p. 847-856.
5. Navaneethan, S.D., et al., *Pragmatic Randomized, Controlled Trial of Patient Navigators and Enhanced Personal Health Records in CKD.* Clin J Am Soc Nephrol, 2017. **12**(9): p. 1418-1427.
6. Peralta, C.A., et al., *Electronic Decision Support for Management of CKD in Primary Care: A Pragmatic Randomized Trial.* Am J Kidney Dis, 2020. **76**(5): p. 636-644.
7. Rifkin, D.E., et al., *Linking clinic and home: a randomized, controlled clinical effectiveness trial of real-time, wireless blood pressure monitoring for older patients with kidney disease and hypertension.* Blood pressure monitoring, 2013. **18**(1): p. 8-15.
8. Samal, L., et al., *Impact of Kidney Failure Risk Prediction Clinical Decision Support on Monitoring and Referral in Primary Care Management of CKD: a Randomized Pragmatic Clinical*

Trial. Kidney medicine, 2022. **4**(7): p. 100493.
9. Sarker, M.H., *A CKD Awareness Campaign and mHealth Education to Improve Knowledge and Quality of Life Among CKD Patients in Bangladesh: a Randomized Controlled Trial.* Journal of the American Society of Nephrology : JASN, 2022. **33**: p. 48-49.
10. Sequist, T.D., et al., *Physician and patient tools to improve chronic kidney disease care.* American journal of managed care, 2018. **24**(4): p. e107-e114.
11. St-Jules, D.E., et al., *An Evaluation of Alternative Technology-Supported Counseling Approaches to Promote Multiple Lifestyle Behavior Changes in Patients With Type 2 Diabetes and Chronic Kidney Disease.* Journal of renal nutrition, 2023. **33**(1): p. 35-44.
12. van Gelder, V.A., et al., *Web-based consultation between general practitioners and nephrologists: a cluster randomized controlled trial.* Family practice, 2017. **34**(4): p. 430-436.

Supplementary Table S1. Eligibility Criteria for Study Inclusion

	Inclusion	Exclusion
Participant	Adults with chronic kidney disease	Patients on dialysis with CKD and patients without CKD
Intervention	Digital health intervention	Without digital health intervention
Comparator	Usual or standard care	Comparators that incorporated digital health intervention
Outcome	Report improvements in desired care	Not reporting improvements in desired care
Study design	Clinical trials	Observational, cross-sectional, pre/post, case studies, case series, case-control, systematic reviews, meta-analysis
Language	English	Non-English
Published date	2009 to 30/6/2023	Before 2009
Publication type	Research article reporting findings	Conference abstract, protocol, commentary, animal study, unpublished literature, retracted articles

CKD: chronic kidney disease

Supplementary Table S2. Characteristics of Included Studies

Study ID, Country	Users of Interventions	Types of interventions	Study design	Total no. of patients (no. of clusters)	Comparator	Outcomes	
						primary	secondary
**Abdel-Kader 2011, the US**	primary care providers	Automated Clinical Reminders	cluster RCT	248 (30)	usual care with education	referral to a nephrologist	albuminuria/proteinuria assessment, CKD documentation, optimal blood pressure, use of renoprotective medications
**Bhardwaja 2011, the US**	providers (pharmacist)	Clinical Decision Support	RCT	6125	usual care	the proportion of medication errors	RE-AIM
**Blakeman 2014, the UK**	patients	an intervention to provide information and telephone-guided access to community support	RCT	436	usual care	health related quality of life (EQ-5D health questionnaire), BP control, positve and active engagement in life (heiQ) at 6 months	an additional five domains of the heiQ , SDSCA, HADS-A, BIPQ, four physical and psychological well-being health education outcome , cost-effectiveness analysis

**Humalda 2020, the Netherlands**	patients	web-based	RCT	94	routine care	sodium excretion after the 3-month intervention and after the 6-month maintenance period	blood pressure, proteinuria, costs, quality of life, self-management skills, and barriers and facilitators for implementation
**Navaneethan 2017, the US**	patients	PHR, CKD alerts	RCT (2*2 factorial design)	107	usual care	ΔeGFR over the 2-year study period	acquisition of appropriate laboratory measures (hemoglobin [Hb], phosphorus, 25-hydroxy vitamin D, parathyroid hormone, LDL cholesterol, HbA1c, and urine albumin-to-creatinine ratio); prescription of renoprotective medications, such as angiotensin-converting enzyme inhibitors(ACEIs) and angiotensin receptor blockers (ARBs); referral rates to nephrologists and vascular surgeons and for kidney transplantation assessment; achieving BP control (,130/80 mmHg); number of hospitalizations and emergency room visits; death

**Peralta 2020, the US**	primary care providers	eCDSS with (pharmacist counseling)	cluster RCT	353 (52)	usual care	△ blood pressure over 12 months	PCP awareness of CKD, use of ACEi/ARB and statin
**Rifkin 2013, the US**	patients	telemonitoring, wireless device	RCT	43	usual care	improved data exchange and device acceptability	BP change
**Samal 2022, the US**	providers & clinics	CDS application	block RCT	5590	usual care	Urine and serum laboratory monitoring test findings measured at one timepoint 6 months after the initial primary care visit	nephrology referral in patients at one timepoint 6 months after the initial primary care visit.

**Sarker 2022, Bangladesh**	patients	mHealth education	parallel-group RCT	126	standard treatment	the evaluation of improved scores on the CKD knowledge questionnaire	QOL and changes in the levels of blood pressure (BP), BMI, serum creatinine, fasting blood sugar (FBS), hemoglobin, cholesterol, high-density lipoprotein cholesterol, triglyceride, serum uric acid, blood urea nitrogen (BUN), and albumin-to-creatinine ratio
**Sequist 2018, the US**	physicians & patients	EHR tools (Alerts) & patient engagement (educational mailings)	Cluster RCT	7691 (153)	no EHR alerts & no mailed outreach program	high-risk patients: an office visit to a nephrologist and the prescription of ACEI/ARB during the 12-month study period Low-risk patients: the presence of urine protein test during the 12-month study period and the prescription of ACEI/ARB	rates of annual serum creatinine, LDL cholesterol, hemoglobin, phosphorus, 25-OH vitamin D, calcium, and parathyroid hormone testing.

**St-Jules 2023, the US**	patients	mobile self-monitoring	RCT (2*2 factorial design)	128	ADVICE group (monthly education handouts)	height and weight	SBP, DBP, and carotid-femoral pulse wave velocity (cf-PWV) blood samples (HbA1c, TC, triglycerides, HDLC, and derived LDLC)
**vanGelder 2017, the Netherlands**	providers	Web-based consultation	cluster RCT	3004 (47)	enhanced usual care	referral rate	consultation rates, QoC, Process indicators disease progression (assessment of eGFR or serum creatinine, albuminuria, glucose and blood pressure); (2) metabolic parameters [assessment of hemoglobin, calcium, phosphate, parathyroid hormone (PTH), serum albumin and potassium]; costs; questionnaires

CKD: chronic kidney disease; RE-AIM: reach, effectiveness, adoption, implementation, maintenance; SDSCA: the Summary of Diabetes SelfCare Activities Measure; HADS-A: Hospital Anxiety and Depression Scale; BIPQ: Brief Illness Perception Questionnaire; PHR: personal health records; LDL: Low Density Lipoprotein; HbA1c: hemoglobin A1c; BMI: body mass index; eGFR: estimated glomerular filtration rate; mHealth: mobile health; CDSS: clinical decision support system; EHR: electronic health records; QOL: quality of life; QoC:quality of care; PCP: primary care practitioner; ACEI: angiotensin-converting enzyme inhibitor;ARB: angiotensin receptor blocker.

Supplementary Figure S1. Risk of Bias Report of Included Studies

RCT	D1a	D1b	D2	D3	D4	D5	Overall
Bhardwaja (2011)	+		+	+	+	+	+
Blakeman (2014)	!		+	+	-	+	-
Humalda (2020)	+		+	+	!	+	!
Navaneethan (2017)	+		+	+	+	+	+
Rifkin (2013)	+		!	+	!	+	!
Samal (2022)	+		-	+	!	-	-
Sarker (2022)	+		+	+	!	+	!
St-Jules (2023)	!		-	+	+	+	-
**cluster RCT**							
Abdel-Kader (2011)	+	+	+	+	+	+	+
Peralta (2020)	!	+	+	+	+	+	!
Sequist (2018)	!	+	!	+	+	+	!
vanGelder (2017)	+	+	!	+	+	+	+

+	Low risk
!	Some concerns
-	High risk

Risk of bias report of included studies using Cochrane's Rob2.
RCT=randomised clinical trials; D1a=Randomisation process; D1b=Timing of identification or recruitment of participants; D2=Deviations from the intended interventions; D3=Missing outcome data; D4=Measurement of the outcome; D5=Selection of the reported result.

Supplrmentary Figure S2a. Abosolute Improvements in Referral Rate.

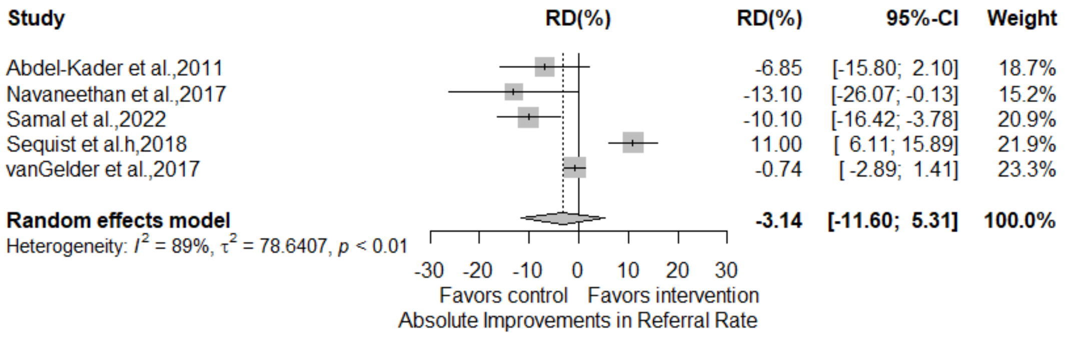

Supplementary Figure S2b. Abosolute Improvements in Test Rate

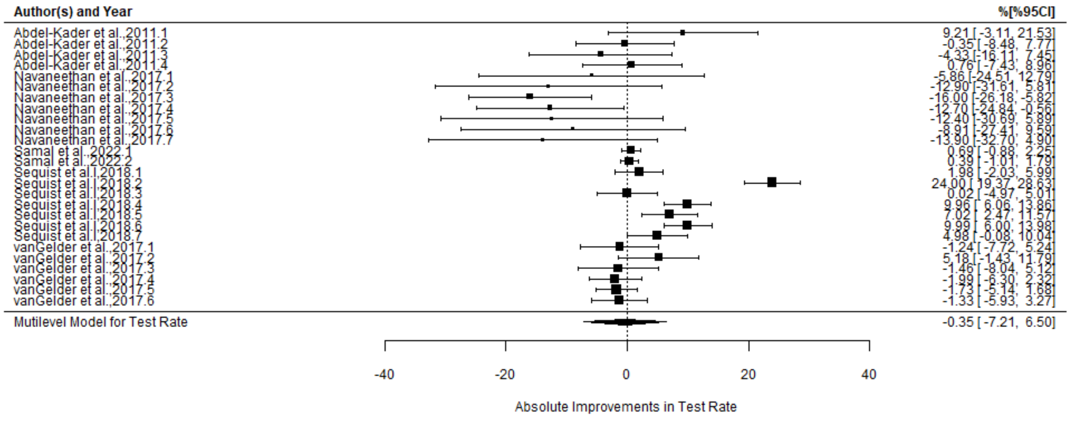

Supplementary Figure S2c. Abosolute Improvements in CKD Documentation

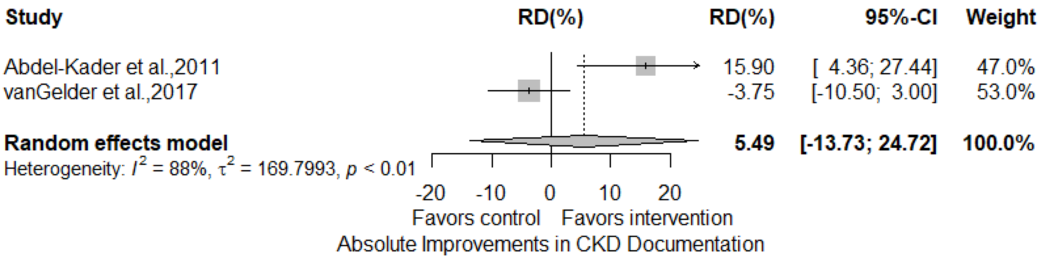

Supplementary Figure S2d. Abosolute Improvements in Medication Use

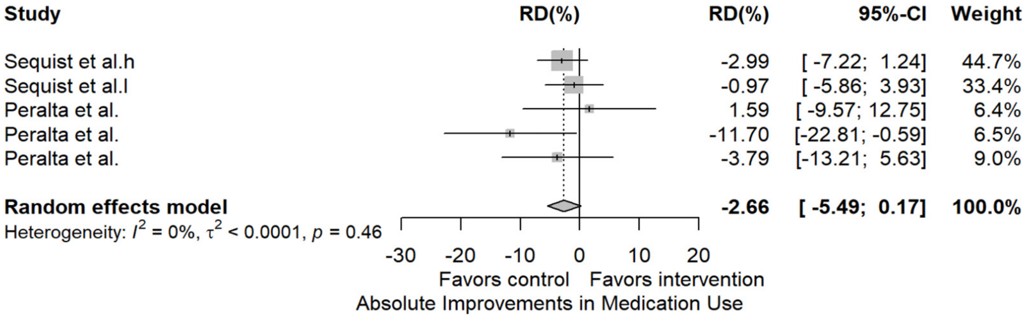

Supplementary Figure S2e. Absolute Improvement of processes of care

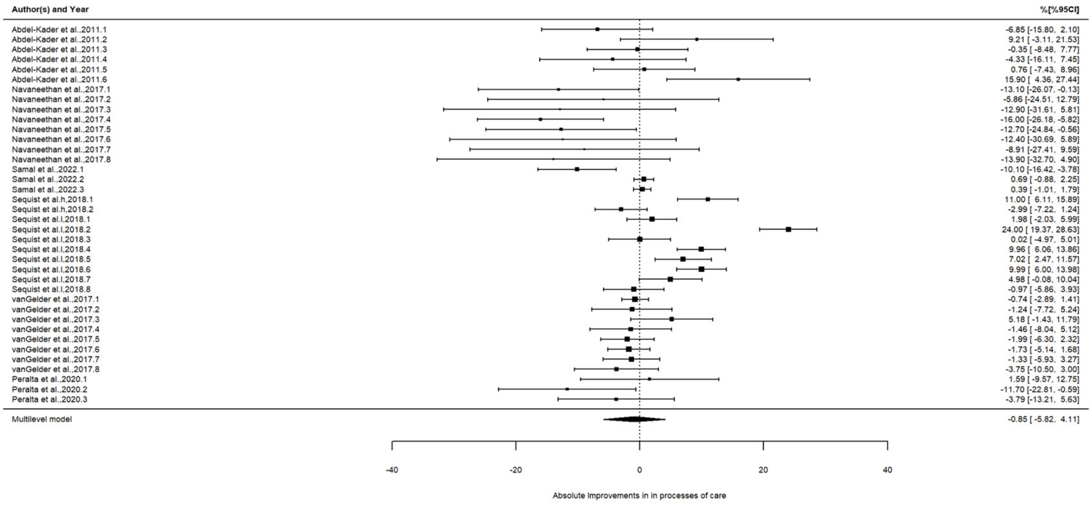

Supplement: Supplementary file 1 [file jcm-13-00364-s001.zip › jcm-2715831-supplementary.pdf]
